# ARHGAP10, downregulated in ovarian cancer, suppresses tumorigenicity of ovarian cancer cells

**DOI:** 10.1038/cddis.2015.401

**Published:** 2016-03-24

**Authors:** N Luo, J Guo, L Chen, W Yang, X Qu, Z Cheng

**Affiliations:** 1 Department of Gynecology & Obstetrics, Yangpu Hospital, School of Medicine, Tongji University, Shanghai 200090, China; 2 Institute of Gynecological Minimally Invasive Medicine, School of Medicine, Tongji University, Shanghai 200090, China

## Abstract

Rho GTPase-activating proteins (RhoGAPs) are implicated in the development and progression of ovarian cancer. ARHGAP10 is a member of RhoGAP proteins and inactivates Cdc42 by converting GTP-bound form to GDP-bound form. Here, we aimed to evaluate ARHGAP10 expression profile and functions in ovarian cancer. The decreased expression of ARHGAP10 was found in 77.3% (58/75) of ovarian cancer tissues, compared with their non-tumorous counterparts. Furthermore, overall survival in ovarian cancer patients with higher expression of ARHGAP10 was longer than those with lower expression. Ectopic expression of ARHGAP10 in two ovarian cancer cell lines with lower expression of ARHGAP10 (A2780 and HO-8910) dramatically suppressed cell proliferation *in vitro*. In nude mice, its stable overexpression significantly inhibited the tumorigenicity of A2780 cells. We further demonstrated that overexpression of ARHGAP10 significantly inhibited cell adhesion, migration and invasion, resulted in cell arrest in G1 phase of cell cycle and a significant increase of apoptosis. Moreover, ARHGAP10 interacted with Cdc42 and overexpression of ARHGAP10 inhibited the activity of Cdc42 in A2780 cells. Gene set enrichment analysis on The Cancer Genome Atlas dataset showed that KEGG cell cycle, replication and base excision repair (BER) pathways were correlatively with the ARHGAP10 expression, which was further confirmed in ovarian cancer cells by western blotting. Hence, ARHGAP10 may serve as a tumor suppressor through inactivating Cdc42, as well as inhibiting cell cycle, replication and BER pathways. Our data suggest an important role of ARHGAP10 in the molecular etiology of cancer and implicate the potential application of ARHGAP10 in cancer therapy.

Rho GTPases act as key regulators of various cell functions, including cell cytoskeleton organization, migration, gene transcription, adhesion, cell proliferation and survival.^1^ ARHGAP10 (also known as ARHGAP21) is an important member of Rho GTPase-activating proteins (RhoGAP),^2^ which catalyze the conversion of active GTP-bound Rho GTPases to the inactive GDP-bound form, and thus suppress Rho GTPases-mediated cellular processes. In addition to its RhoGAP domain, ARHGAP10 contains a PDZ and a pleckstrin homology domain. Besides serving as a RhoGAP for Cdc42,^3^ RhoA and RhoC,^4^ ARHGAP10 can also interact with several proteins, including *α*-catenin,^5^ ARF1,^3^ FAK, PKC-*ζ*^6^ and *β*-arrestin 1,^7^ and thus is involved in various cell functions, such as cell junction formation,^5^ vesicular trafficking of Golgi membranes,^3^ cardiac stress,^6^ influenza virus replication^8^ and stress fiber formation.^7^ Recently, methylation and single nucleotide polymorphisms of ARHGAP10 have been described in pediatric leukemia^9^ and invasive breast cancer,^10^ respectively, which indicated the association of ARHGAP10 and cancer.

Ovarian cancer is the most lethal gynecologic malignancy.^11^ In spite of advances in surgery, radiation and chemotherapy, the overall survival of ovarian cancer patients remains poor, with a 5-year survival rate of merely 30%.^12^ The main reasons for high mortality of this disease appear to be to the late clinical presentation and the high rate of recurrence.^13^ In order to improve early detection and develop new therapeutics of ovarian cancer, a better understanding of the molecular mechanisms underlying the carcinogenesis of ovarian cancer is needed. A series of recent studies has shown an association between ARHGAP10 and various cancers, such as head and neck squamous cell carcinoma,^14^ gliomas^15^ and prostate cancer.^4^ Emerging evidence has liked other RhoGAPs to the development and progression of ovarian cancer.^16, 17^ Here, we aimed to evaluate ARHGAP10 expression and functions in ovarian cancer.

In the present study, we compared ARHGAP10 expression between ovarian cancer and paired normal tissues. The effects of ARHGAP10 overexpression in the proliferation, adhesion, migration and invasion of ovarian cancer cells were then assessed. The involved possible mechanisms were also explored. Our study provides the evidences that ARHGAP10 expression is decreased in ovarian cancer, and it may be a prognosis factor and tumor suppressor for this disease.

## Results

### Reduced expression of ARHGAP10 was correlated with poor prognosis of ovarian cancer

**Figure 1:**
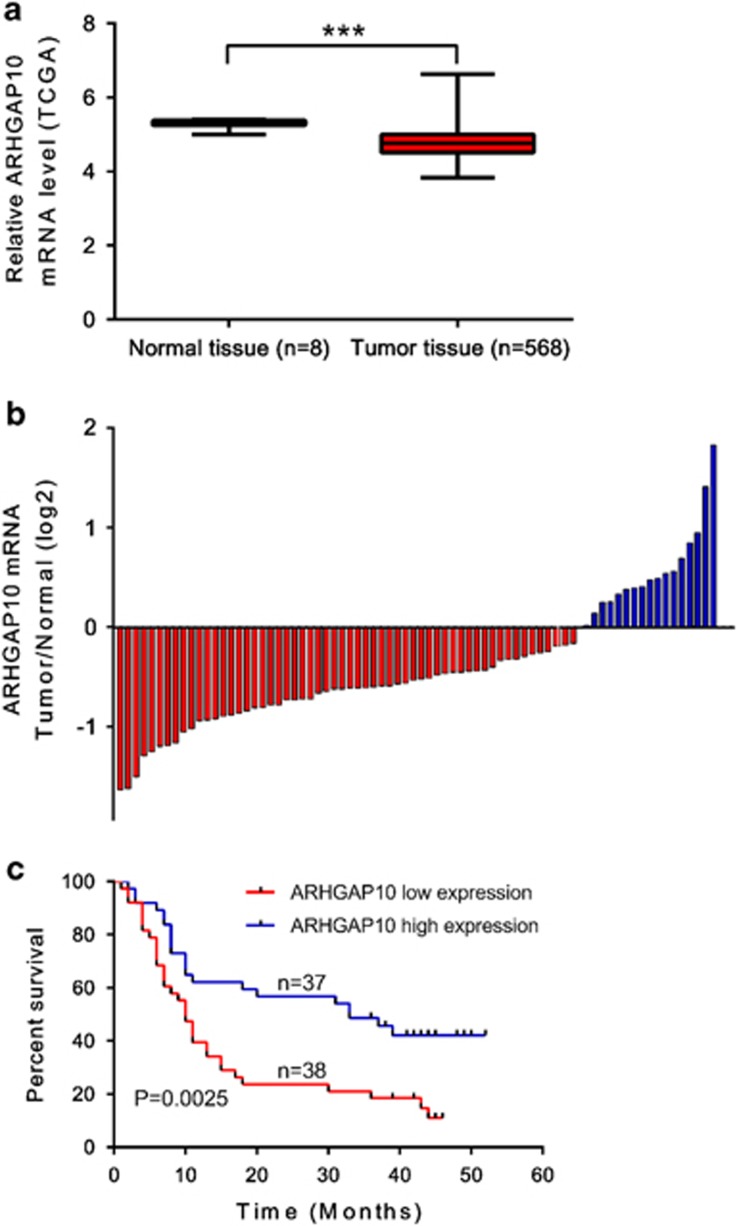
ARHGAP10 expression in human ovarian cancer tissues. (**a**) ARHGAP10 was downregulated in ovarian cancer tissue compared with the normal tissue in TCGA dataset. (**b**) Relative expression of ARHGAP10 in ovarian cancer tissues (*n*=75) compared with corresponding non-tumor tissues (*n*=75). ARHGAP10 expression was examined by real-time PCR and normalized to GAPDH expression. Results are presented as log2 (tumor/normal). (**c**) The correlation between ARHGAP10 expression and prognosis

We first re-analyzed microarray-based gene expression data downloaded from The Cancer Genome Atlas (TCGA, ovarian serous cystadenocarcinoma cohort, https://tcga-data.nci.nih.gov/tcga/) and found that ARHGAP expression was significantly decreased in ovarian tissue compared with the normal tissue from individuals who did not have cancer but were able to donate tissue for other reasons (*P*<0.001). We then detected mRNA levels of ARHGAP10 in 75 pairs of ovarian cancer and adjacent non-tumorous epithelial tissues by quantitative real-time PCR. The log2 (tumor/normal) of each tumor sample was then calculated. Positive log2 (Tumor/Normal) indicated increased expression of ARHGAP10 in tumor tissue while negative log2 indicated reduced expression of ARHGAP10 in tumor tissue. As shown in Figure 1b, ARHGAP10 expression was decreased in 77.3% (58/75) of tested ovarian cancer tissues. Statistical analysis with student's *t*-test suggested that ARHGAP10 expression was significantly downregulated in ovarian cancer tissues compared with that in noncancerous tissues (*P*<0.0001).

Then, according to the relative ARHGAP10 expression in tumor tissues, the 75 ovarian cancer patients were classified into two groups: relative high group (*n*=37) and relative low group (*n*=38) by using a value of 1.16 (median) as a cutoff. Kaplan–Meier analysis was performed to investigate the correlation of ARHGAP10 expression and prognosis. As shown in Figure 1c, the overall survival time of patients with high ARHGAP10 expression was notably longer than those with low ARHGAP10 expression (*P*<0.01). Our data demonstrated that ARHGAP10 expression was downregulated in ovarian cancer tissues, which was correlated with poor survival of patients with ovarian cancer.

### Ectopic overexpression of ARHGAP10 inhibited cell proliferation of ovarian cancer cells *in vitro* and *in vivo*

**Figure 2:**
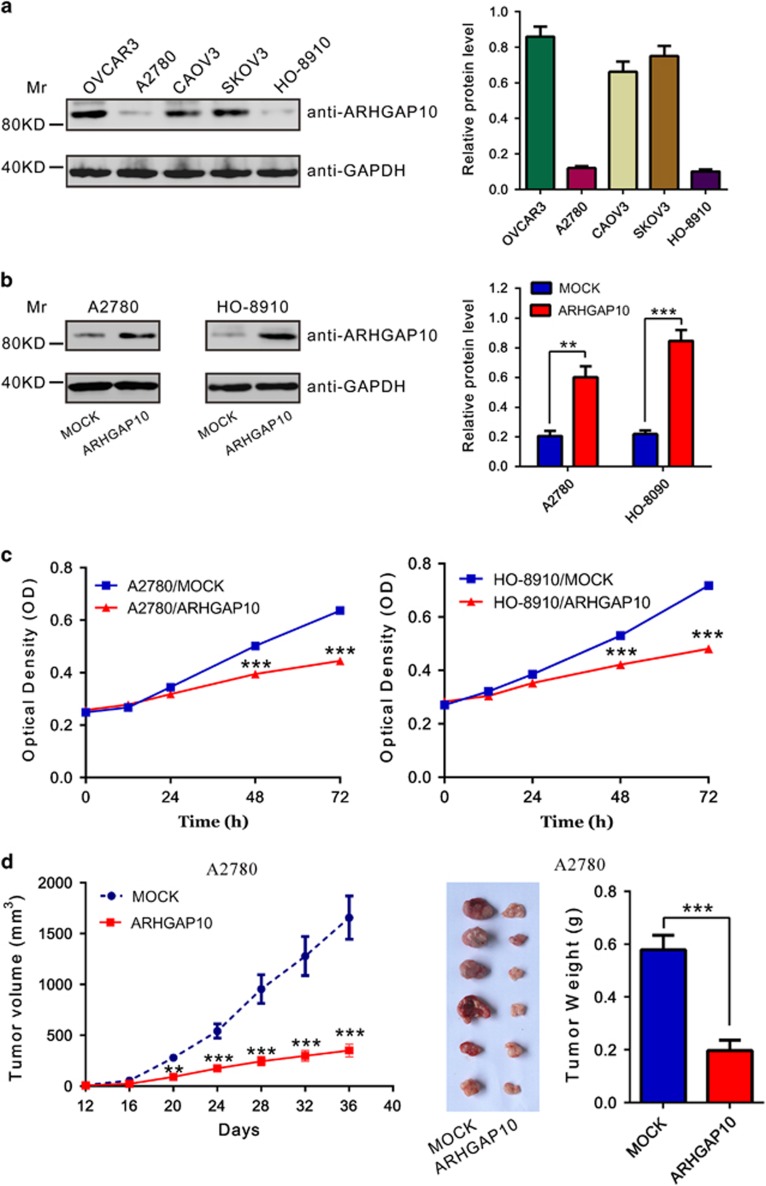
Knockdown ARHGAP10 inhibited ovarian cancer cells proliferation *in vitro* and *in vivo*. (**a**) Expression of ARHGAP10 in five ovarian cancer cell lines as determined by western blotting. Left panel, representative results of western blot; right panel, protein levels relative to GAPDH. (**b**) Ectopic expression of ARHGAP10 in A2780 and HO-8910 cells was detected by western blotting. (**c**) Ectopic expression of ARHGAP10 significantly reduced cell proliferation as determined by CCK-8 assay. (**d**) A2780 cells stably infected with vector or ARHGAP10 virus were subcutaneously inoculated into nude mice (six per group). Overexpression of ARHGAP10 significantly inhibited tumor growth in nude mice xenograft model (**P<0.01, ***P<0.001)

ARHGAP10 expression was then estimated in five ovarian cancer cell lines, OVCAR3, A2780, CAOV3, SKOV3 and HO-8910, by western blotting. Two cell lines, A2780 and HO-8910, showed lower level of ARHGAP10, while the other three cell lines showed higher level (Figure 2a).

To investigate the function of ARHGAP10 in ovarian cancer cells, A2780 and HO-8910 stable pool cells were established by infected with vector control (MOCK) or ARHGAP10 lentivirus and puromycin selection. The ectopic expression of ARHGAP10 in both cells was confirmed by western blotting (Figure 2b).

To determine the effect of ARHGAP10 on cell proliferation, we monitored the proliferation rate of ARHGAP stable expressing cells (A2780/ARHGAP10 and HO-8910/ARHGAP10) for 3 days by CCK-8 assay. We observed that A2780/ARHGAP10 and HO-8910/ARHGAP10 cells exhibited a significantly slower growing phenotype than corresponding control cells (MOCK, Figure 2c). These results indicated that ARHGAP10 exerts growth-inhibitory effects on ovarian cancer cells.

To determine the effect of ARHGAP10 on tumorigenicity *in vivo*, equal number of A2780 stably expressed vector (MOCK) or ARHGAP10 was injected subcutaneously into nude mice and tumor formation was examined for 36 days. As shown in Figure 2d, although both cells were able to form tumors, the tumor growth rate of nude mice injected with ARHGAP10-overexpressed cells was significantly more slowly than that of mice injected with control cells. The volume and weight of ARHGAP10-overexpressed tumors was less than 35% that of control tumors at 36 days when the nude mice were killed.

### ARHGAP10 overexpression induced G1 phase arrest and cell apoptosis

**Figure 3:**
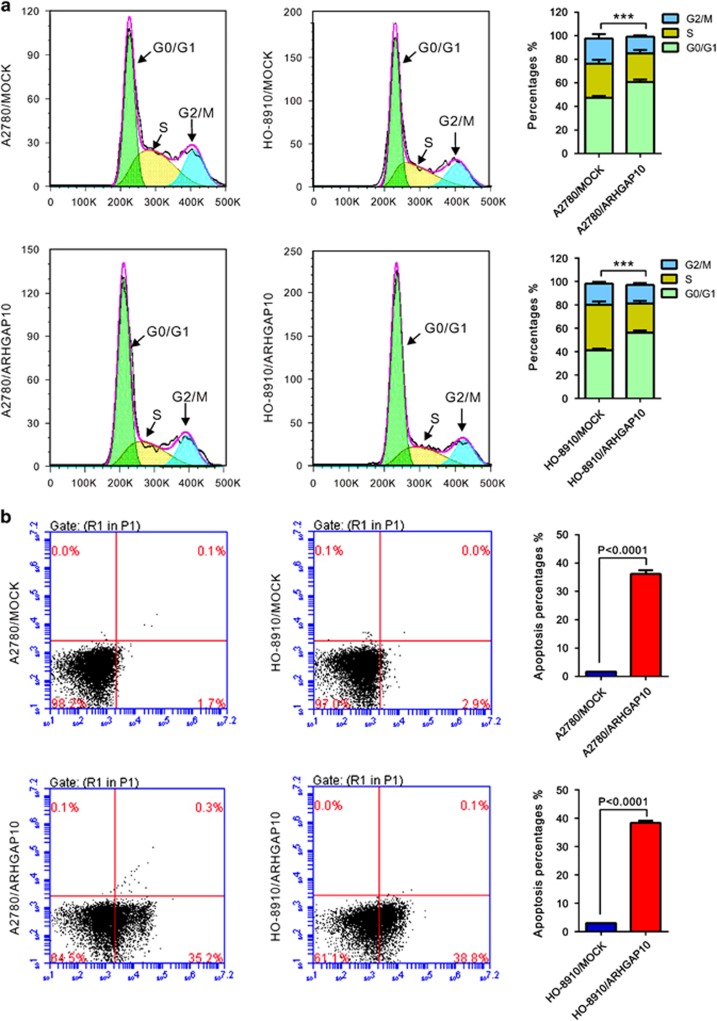
ARHGAP10 overexpression induced cell arrest in G1 phase of cell cycle (**a**) and a dramatic increase of apoptosis (**b**). Data were based on at least three independent experiments, and shown as mean±S.D. (***P<0.001)

Then we assessed whether ARHGAP10 affects the cell cycle of ovarian cancer cells by propidium iodide (PI) staining and flow cytometry analysis. As shown in Figure 3a, compared with MOCK cells, ARHGAP10 overexpression led to a significant increase of G0/G1 phase cells (increased ratio: A2780, 28.4% HO-8910, 36.2%).

We then explored the effects of ARHGAP10 on cell apoptosis by Annexin V-fluorescein isothiocyanate/PI staining assay. As shown in Figure 3b, a notable increase of cell apoptosis was noted in ARHGAP10-overexpressed A2780 and HO-8910 cells as compared with MOCK cells. These data suggested that increased expression of ARHGAP10 notably induced G0/G1 phase cell cycle arrest and cell apoptosis of ovarian cancer cells, which may cause the inhibition of cell proliferation.

### ARHGAP10 overexpression inhibited the adhesion, motility and invasiveness of ovarian cancer cells

**Figure 4:**
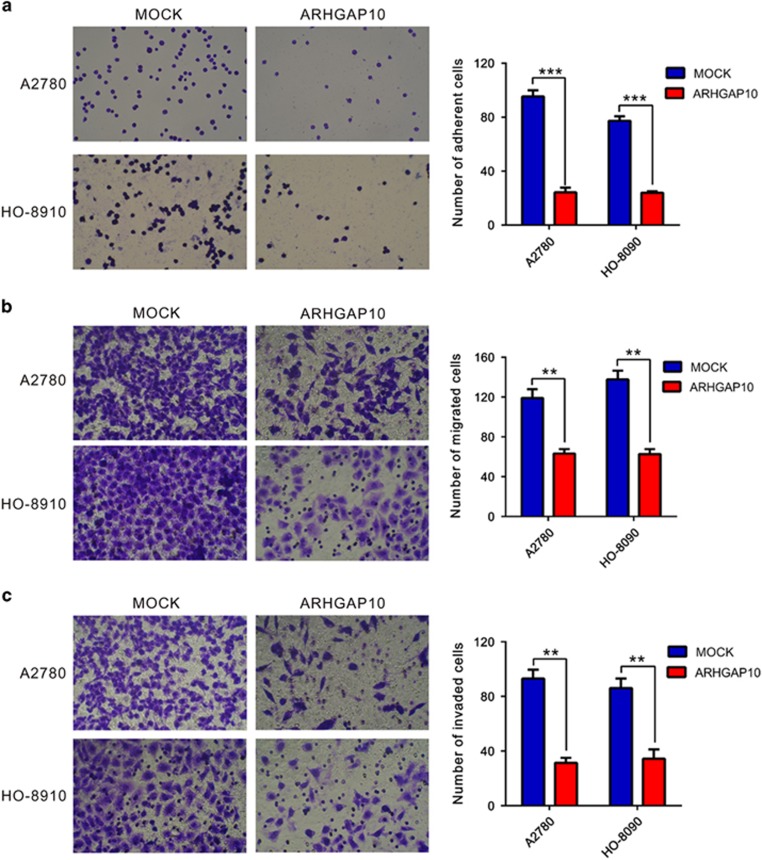
ARHGAP10 overexpression inhibited the adhesion, motility and invasiveness of ovarian cancer cells. Quantitative cell adhesion (**a**), migration (**b**) and invasion assay (**c**) of cells transfected with vector (MOCK) or ARHGAP10 (**P<0.01, ***P<0.001)

Tumor cells are often characterized by the reduction in cell–cell and/or cell–matrix adhesion, which correlates with tumor invasion and metastasis.^18^ Then, we investigated whether ARHGAP10 affected the adherent, migrated and invasive ability of ovarian cancer cells by fibronectin-adhesion assay and Transwell assay, respectively. As shown in Figure 4, ARHGAP10-overexpressed cells have lower adherent ability than MOCK cells (Figure 4a). ARHGAP10 overexpression caused a significant reduction in cell migration, with only 52.9% and 39.5% cells migrating in A2780 and HO-8910 cells, respectively (Figure 4b). ARHGAP10-overexpressed cells showed significant reduced invasive ability compared with control cells. The number of invaded cells was 33.3% and 39.5% of that of the control cells in A2780 and HO-8910 cells, respectively (Figure 4c). These data suggested an inhibitory role of ARHGAP10 on ovarian cancer metastasis.

### ARHGAP10 inhibited the activation of Cdc42

**Figure 5:**
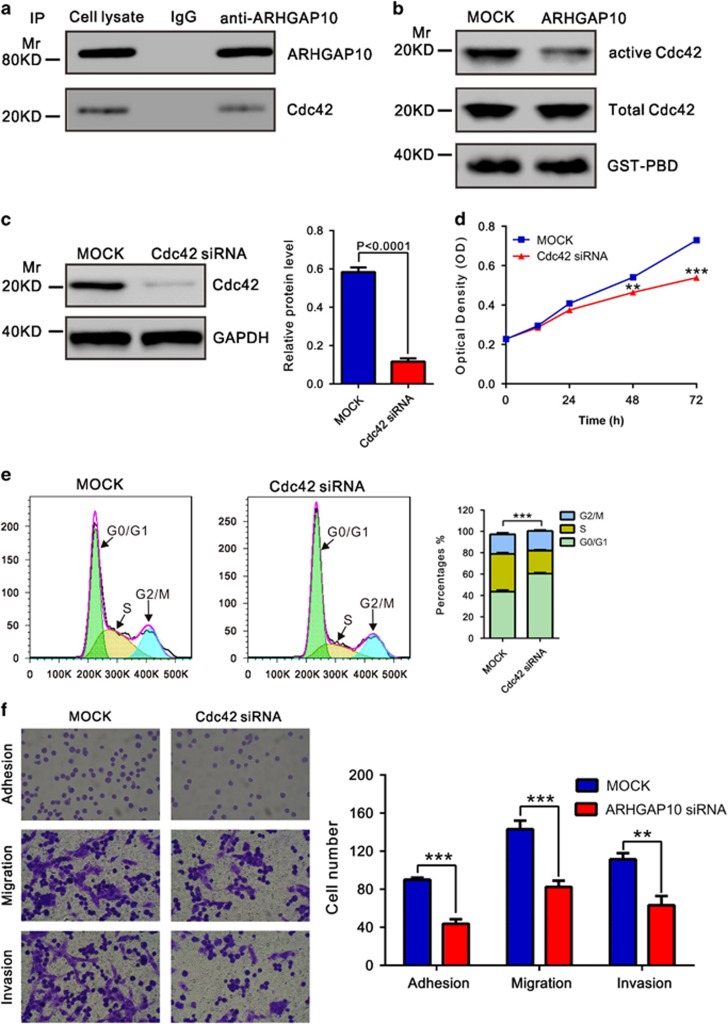
ARHGAP10 functions through inhibiting Cdc42 activation. (**a**) Co-immunoprecipitation analysis showed that ARHGAP10 interacts with Cdc42 in A2780 cells. (**b**) Activation of Cdc42 was determined by GST-PBD pull-down assay. GST-PBD fusion protein was used as quantitative control, and total Cdc42 in protein lysates was shown. (**c**) Cdc42 expression was detected in A2780 cells transfected with Cdc42 siRNA or control siRNA (Mock) by western blotting. (**d**) Cdc42 knockdown significantly reduced cell proliferation as determined by CCK-8 assay. (**e**) Cdc42 knockdown induced cell arrest in G1 phase of cell cycle. (**f**) Cdc42 knockdown notably inhibited the adhesion, motility and invasiveness of A2780 cells (**P<0.01, ***P<0.001)

ARHGAP10 has been shown to be a potent GAP for Cdc42.^3^ We then tried to detect their interaction by co-immunoprecipitation experiments in A2780 cells. As shown in Figure 5a, the endogenous Cdc42 co-immunoprecipitated with ARHGAP10, but this did not occur with the control IgG. Moreover, GST-PAK1-binding domain pull-down assay was carried out to test Cdc42 activity followed by western analysis with an anti-Cdc42 antibody. As shown in Figure 5b, ectopic expression of ARHGAP10 notably inhibited the activity of Cdc42, while Cdc42 expression was not affected by ARHGAP10. Cdc42 has been implicated in the promotion of tumorigenesis.^19^ We then suppressed its expression in A2780 cells (Figure 5c) and found that Cdc42 knockdown significantly suppressed the proliferation, cell cycle progression, adhesion, motility and invasiveness of A2780 cells. These data suggested that ARHGAP10 may exert its function in ovarian cancer through suppressing Cdc42 activity.

### ARHGAP10-associated pathways in ovarian cancer

**Figure 6:**
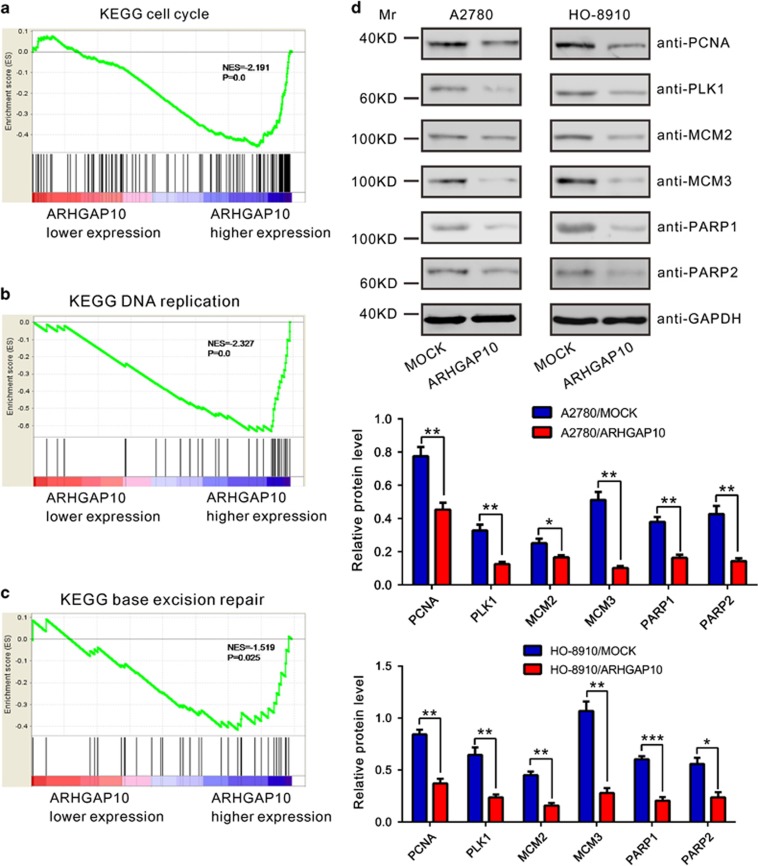
ARHGAP10-associated pathways in ovarian cancer. (**a**–**c**) GSEA was performed using TCGA ovarian serous cystadenocarcinoma dataset. The KEGG cell cycle, DNA replication and BER pathways were identified with the strongest association with ARHGAP10-lower expression. (**d**) Key moderators were determined by western blotting in A2780 and HO-8910 cells. Upper panel, representative results of western blotting; middle and lower panel, protein levels relative to GAPDH. Data were based on at least three independent experiments, and shown as mean±S.D. (*P<0.05, **P<0.01, ***P<0.001)

To further probe the ARHGAP10-associated pathways on an unbiased basis, we performed Gene Set Enrichment Analysis (GSEA) using data of the OV cohort from TCGA (568 patients). GSEA is designed to detect coordinated differences in expression of predefined sets of functionally related genes.^20^ The KEGG cell cycle, DNA replication and base excision repair (BER) pathways were identified with the significant association with ARHGAP10 expression in the TCGA dataset (Figure 6a and c).

To validate the GSEA results, we then detected the expression of cell cycle (PCNA and PLK1), DNA replication (MCM2 and MCM3) and BER pathways-related proteins (PARP1 and PARP2) in ovarian cancer cells transiently overexpressed ARHGAP10. The levels of detected protein were significantly decreased in both A2780 and HO-8910 cells (Figure 6d) after the overexpression of ARHGAP10.

## Discussion

ARHGAP10 belongs to RhoGAP proteins, which negatively regulate Rho GTPases. The roles of RhoGAP proteins have been investigated in cancer development and progression.^21, 22, 23, 24, 25^ Involvement of ARHGAP10 in cancers has also been recently concerned.^4, 14, 15^ In the present study, we confirmed that ARHGAP10 was extremely downregulated in 75 ovarian cancer tissues, compared with paired normal tissues. Importantly, the overall survival of patients with lower ARHGAP10 expression levels was shorter than that with higher expression. These findings indicate that ARHGAP10 may be considered as a novel prognostic marker for ovarian cancer.

To further investigate the functions of ARHGAP10 in ovarian cancer, we explored the effects of ARHGAP10 overexpression on the cell behavior of ovarian cancer cells. Ectopic expression of ARHGAP10 in two ovarian cancer cell lines resulted in a significant inhibition in cell proliferation (Figure 2), adhesion, migration and invasion (Figure 4), and a remarkable induction in cell cycle G1 phase arrest and apoptosis (Figure 3). Tumorigenicity in nude mice was also suppressed by ARHGAP10 overexpression (Figure 2d). While, downregulation of ARHGAP10 expression by siRNA transfection in higher expression OVCAR3 cells induced cell proliferation, adhesion, migration and invasion (Supplementary Figure S1). Our data indicate that ARHGAP10 may act as a tumor suppressor in ovarian cancer.

Further, we tried to explore the mechanisms by which ARHGAP10 exerts its function. GSEA indicated that ARHGAP10 expression was associated with the KEGG cell cycle, DNA replication and BER pathway (Figure 5). Cell cycle and DNA replication regulation are frequently abnormal in most common malignancies, resulting in aberrant cell proliferation.^26^ Cancer occurs primarily in proliferative tissues. When DNA damage in proliferating cells are not repaired because of inadequate expression of a DNA repair gene, the risk of cancer is increased.^27^ Here, ARHGAP10 overexpression remarkably decreased cell cycle (PCNA^28, 29^ and PLK1^30, 31^), DNA replication (MCM2^32, 33^ and MCM3^34, 35^) and BER pathway (PARP1 and PARP2^36, 37^)-related factors, which may account for its suppression effects on the tumorigenicity of ovarian cancer. Furthermore, Dubois *et al.*^3^ has demonstrated that ARHGAP10 functions as a potential GAP for Cdc42 *in vitro*. Previous studies have demonstrated a role for Cdc42 in promoting cell cycle progression.^38, 39, 40^ Here, we confirmed that ARHGAP10 can bind with Cdc42 (Figure 5a), thus inhibiting Cdc42 activity (Figure 5b). We also found that Cdc42 knockdown significantly inhibited cell proliferation (Figure 5c) and cell cycle progression (Figure 5d). These data suggested that ARHGAP10 might have a pivotal role in regulation of the cell cycle and DNA replication signal pathways through inhibited Cdc42 activation. Further investigations are required to understand the exact mechanisms by which ARHGAP10 regulates these pathways.

In conclusion, our study suggests that ARHGAP10 acts as a tumor suppressor in ovarian cancer cell line, and the downregulation of ARHGAP10 expression is closely associated with the poor prognosis of ovarian cancer. Whether ARHGAP10 can be used as a potential therapeutic target for ovarian cancer remains to be further investigated.

## Materials and Methods

### Patients and tissue samples

Ovarian cancer tissues and paired adjacent noncancerous epithelial tissues were collected from 75 patients diagnosed with Stage II/III epithelial ovarian serous adenocarcinoma, who were admitted to Gynecology & Obstetrics, Yangpu Hospital, Tongji University (Shanghai, China) between 2008 and 2009. Tissue samples were immediately frozen in liquid nitrogen and kept at −80 °C until used. The study was approved by Ethics Committee of Tongji University and written informed consent was obtained from all patients. All patients have complete clinical and pathological follow-up data. Overall survival was defined as the interval between the dates of surgery and death.

### RNA isolation and quantitative RT-PCR

Total RNA was extracted from tissues using Trizol reagent (Invitrogen, Carlsbad, CA, USA) according to the manufacturer's instructions. The mRNA levels of ARHGAP10 were determined by quantitative RT-PCR using the SYBR Green (Thermo Fisher Scientific, Rockford, IL,USA) on ABI 7300 instrument instrument (Applied Biosystems, Foster City, CA, USA), with GAPDH as an internal control. The primers are as follows: ARHGAP10 (NM_024605.3), 5′-ACTGAAACCCTGATTAAACC-3′ and 5′-ATCTGCCTCTTGTAAATGTG-3′ GAPDH (NM_001256799.1), 5′-CACCCACTCCTCCACCTTTG-3′ and 5′-CCACCACCCTGTTGCTGTAG-3′. All reactions were conducted using the following cycling parameters, 95 °C for 10 min, followed by 40 cycles of 95 °C for 15 s, 60 °C for 45 s. Verification of specific product amplification was determined by dissociation curve analysis. Comparative Ct method was used for quantification of the transcripts. The fold-change for target genes normalized by internal control was determined by the formula 2^−△△CT^. All data represent the average of three replicates.

### Cell culture, transfection, lentiviral infection and RNA interference

Five human ovarian cancer cell lines (OVCAR3, A2780, CAOV3, SKOV3 and HO-8910) were obtained from Chinese Type Culture Collection, Chinese Academy of Sciences and maintained at 37 °C in a humidified air atmosphere containing 5% CO_2_. All culture medium (Life Technologies, Grand Island, NY, USA) were supplemented with 10% fetal bovine serum, 100 U/ml penicillin sodium and 100 mg/ml streptomycin sulfate. OVCAR3, A2780 and HO-8910 cells were cultured in RPMI 1640. CAOV3, SKOV3 and HEK 293 T cells were cultured in DMEM.

Lentiviral constructs of CD513B-1 empty vector (System Biosciences, Mountain View, CA, USA) or CD513B-1-ARHGAP10 were cotransfected with viral packaging plasmids (psPAX2 and pMD2.G) into HEK293T cells by using lipofectamine 2000 (Invitrogen) according to the manufacturer's instruction. Viral supernatant was harvested after 48 h and filtered through 0.45-*μ*m filter. A2780 cells and HO-8910 cells were infected with ARHGAP10 lentivirus or control lentivirus in the presence of 8 *μ*g/ml Polybrene. Stable pools were obtained in the presence of 0.5 *μ*g/ml puromycin (Sigma, St. Louis, MO, USA) and used for following assays.

siRNA specific targeting human Cdc42 (GGACGGAUUGAUUCCACAU) and negative control siRNA were synthesized by Genepharma Co., Ltd (Shanghai, China). siRNA transfection was performed on A2780 cells using Lipofectamine2000 (Invitrogen).

### Antibodies and western blotting

Primary antibodies were obtained from the following companies: (i) ARHGAP10 and Cdc42, Santa Cruz Biotech (Santa Cruz, CA, USA); (ii) GAPDH, CST Biotech (Danvers, MA, USA); (iii) PCNA, PLK1, MCM2, MCM3, PARP1 and PARP2, Abcam (Cambridge, MA, USA). Horseradish peroxidase-conjugated goat anti-mouse secondary antibody or goat anti-rabbit secondary antibody was purchased from Beyotime (Shanghai, China).

Cells were lysed in ice-cold radioimmunoprecipitation assay buffer (50 mM Tris-HCl [pH 7.5], 150 mM NaCl, 1% Triton X-100, 0.5% Na-deoxycholate) containing protease inhibitors. Protein concentration was measured by BCA protein assay kit (Thermo Fisher Scientific). Equal amount of cell lysates were separated on SDS-PAGE gels, transferred to PVDF membranes and analyzed by western blotting using enhanced chemiluminescence system (Bio-Rad, Richmond, CA, USA). Band intensities were measured using Image J (NIH, Bethesda, MD, USA) and normalized to GAPDH.

### Immunoprecipitation

For immunoprecipitations, anti-ARHGAP10 was coupled with protein A-Sepharose beads (Sigma) in RIPA buffer for 2 h or overnight at 4 °C. The immune complex was then added to cell lysate and incubated at 4 °C for 2 h. The resulting beads were washed with TBS buffer (50 mmol/l Tris-HCl (pH 7.4), 150 mmol/l NaCl) to eliminate the nonspecific binding three times. After centrifugation, the immunoprecipitated samples were resuspended in Laemmli sample buffer, boiled at 95–100 °C for 5 min, separated on SDS-PAGE gels, transferred to PVDF membranes and analyzed by western blotting.

### GST-PAK1-binding domain pull-down assay

GST-PAK1-binding domain pull-down assay was performed in A2780 stale cells by using Active Cdc42 Pull-Down and Detection Kit (Life Technologies) according to the manufacturer's protocol. The resulting beads were resuspended in Laemmli sample buffer and analyzed via western blotting.

### Cell proliferation assay

Cell proliferation was measured using Cell Counting Kit-8 (CCK-8) Assay Kit (Dojindo Lab, Kumamoto, Japan).^41^ Stable pool cells at a density of 2 × 10^3^ cells per well were seeded onto 96-well plates. At 0, 12, 24, 48 and 72 h, CCK-8 solution was added to each well and incubated for 1 h. Optical density values at wavelength 450 nm were measured by a microplate reader (Bio-Rad). All conditions were tested in six replicates.

### *In vivo* tumorigenicity assay

The animal study was carried out in accordance with the guidelines approved by the Animal Experimentation Ethics Committee of Yangpu Hospital, Tongji University. Athymic Balb/c nude mice (aged 5 weeks) were provided by Slac Laboratory Animal Co. Ltd. (Shanghai, China). The mice were housed in a pathogen-free animal facility and randomly assigned to the control or experimental group (six mice per group). A2780 sable cells were harvested and injected intraperitoneally into the flank of each mouse (2 × 10^6^/0.1 ml). Tumor volume was estimated every 3 days using the following formula: volume=1/2 × length × width^2^. All mice were killed after 36 days.

### Evaluation of cell cycle distribution and cell apoptosis by flow cytometry

PI staining was used to analyze DNA content. Stable pool cells were harvested and fixed with 70% ethanol at −20 °C overnight. After treatment with PI/ribonuclease staining kits (Multisciences, Hangzhou, China), DNA content was analyzed on a flow cytometer (BD Biosciences, Franklin Lakes, NJ, USA). The percentage of cells in the G0/G1, S, and G2/M phases was determined by the FlowJo cell cycle analysis program (Tree Star, San Carlos, CA, USA).

The percentage of cells actively undergoing apoptosis was determined by double staining with Annexin V-fluorescein isothiocyanate and PI. Stable pool cells were harvested, double-labeled with Annexin V-fluorescein isothiocyanate and PI apoptosis detection kits (KeyGEN Biotech, Nanjing, China), and analyzed using a FACScan flow cytometry. At least 20 000 cells were acquired for each sample. The experiments were performed in triplicate.

### Cell adhesion assay

Stable pool cells were seeded into fibronectin pre-coated 12-well plates at a density of 1 × 10^5^ cells per well and allowed to adhere at 37 °C for 1 h. After non-adherent cells were washed off with PBS, attached cells were fixed in 4% paraformaldehyde for 10 min and stained with GIEMSA solution. The adherent cells were counted and imaged under the microscope under an Olympus inverted microscope (Lake Success, NY, USA).

### Transwell assay

To determine cell migration, stable pool cells were plated in medium without serum in the top chamber of a transwell (Corning, Corning, NY, USA). The bottom chamber contained standard medium with 10% fetal bovine serum. After 24-h incubation, the cells that had migrated to the lower surface of the membrane were fixed with formalin, stained with 0.05% crystal violet, counted and imaged under the microscope. Experiments were carried out at least three times. Cell invasion assay was performed in a Matrigel-coated Transwell and other procedure was the same as described above.

### Gene set enrichment analysis (GSEA)

GSEA is a method of analyzing and interpreting microarray and such data using biological knowledge.^42^ In this study, ovarian serous cystadenocarcinoma cohort was obtained from TCGA (https://tcga-data.nci.nih.gov/tcga/) and analyzed by GSEA as previously described.^43, 44, 45^ GSEA firstly generated an ordered list of all genes according to their correlation with ARHGAP10 expression, and then a predefined gene set receives an enrichment score, which is a measure of statistical evidence rejecting the null hypothesis that its members are randomly distributed in the ordered list. The expression level of ARHGAP10 gene was used as phenotype label, and ‘Metric for ranking genes' was set to Pearson Correlation.

### Statistical analysis

Statistical analysis was performed using GraphPad Prism 6 (GraphPad Software). Data were presented as the mean±standard deviation (S.D.). The data were analyzed using the two-tailed Student's *t*-test to calculate the statistical significance of difference between groups. Kaplan–Meier method and log-rank test were performed for patients' survival analyses. Statistically significant differences were defined as having a *P*<0.05.

## Abbreviations

RhoGAP: Rho-GTPase-activating proteins
TCGA: The Cancer Genome Atlas
CCK-8: Cell Counting Kit-8
PI: propidium iodide
PBD: PAK1-binding domain
GSEA: Gene set enrichment analysis
S.D.: standard deviation
BER: base excision repair
