# Supplementary material for: ARHGAP10, downregulated in ovarian cancer, suppresses tumorigenicity of ovarian cancer cells
Source: Cell Death Dis. 2016 Mar 24;7(3):e2157–. doi: 10.1038/cddis.2015.401 (PMC4823924; doi:10.1038/cddis.2015.401)
Supplement: Supplementary Figure 1 [file cddis2015401x1.docx]

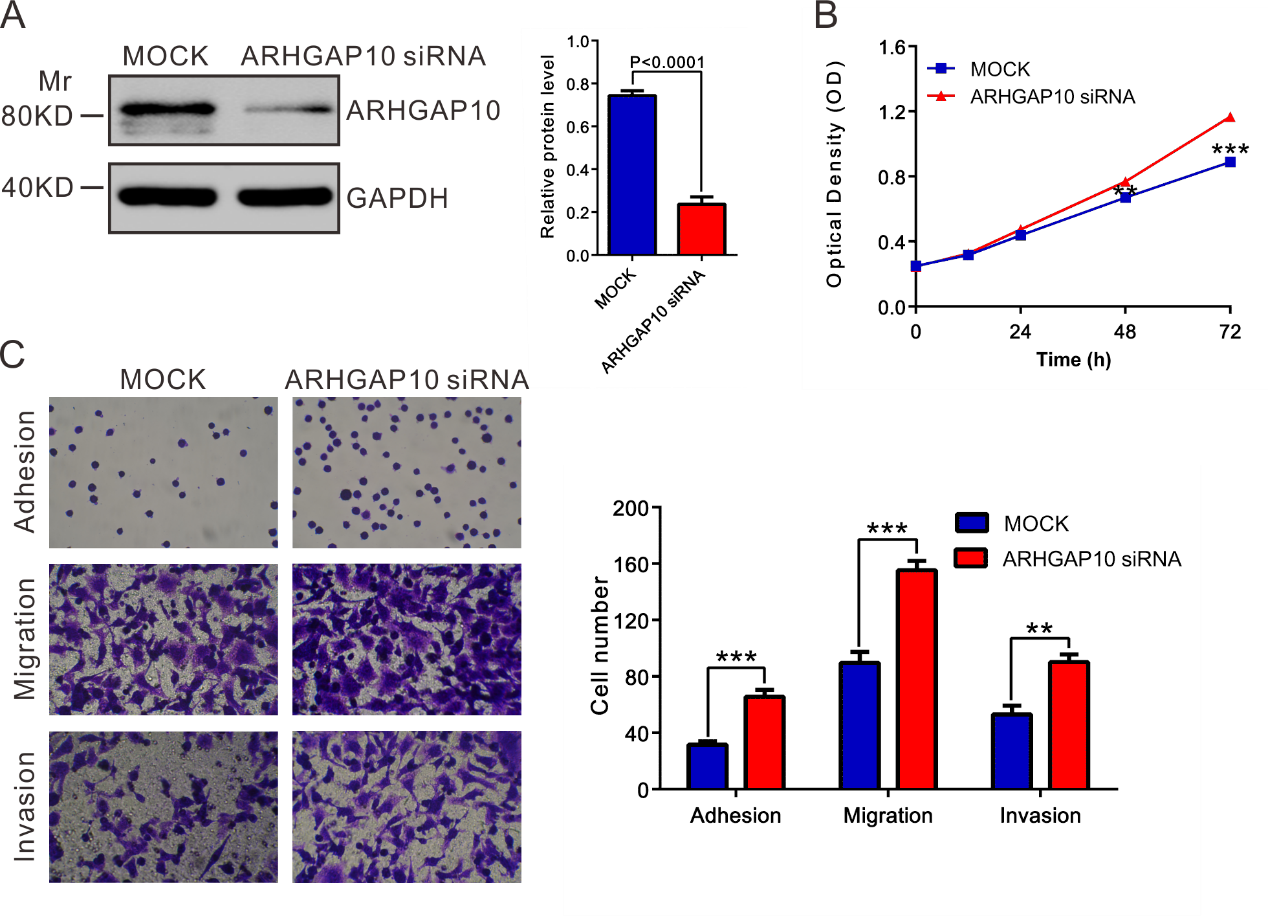


**Figure S1.** Effects of ARHGAP10 knockdown on the proliferation, adhesion, migration and invasion of OVCAR3 cells. ARHGAP10 siRNA (GAACACAGCUCGGAAUUAU) and control siRNA (Mock) were synthesized and transfected into OVCAR3 cells. ARHGAP10 knockdown resulted in a significant decrease in ARHGAP10 protein expression (A), and an increase in cell proliferation (B), adhesion, migration and invasion of OVCAR3 cells (C). (***P*<0.01, ****P*<0.001).
